# Exploring the coupling mode of water and fertilizer for improving growth, fruit quality, and yield of the pear in the arid region

**DOI:** 10.1515/biol-2022-0911

**Published:** 2024-07-23

**Authors:** Tianle Li, Zhijian Gao, Xinlu Bai, Sihai Yu, Shijie An, Qiangqing Zheng, Zhihui Tang, Jinhu Zhi

**Affiliations:** College of Agriculture, Tarim University, Alar, 843300, China; Research Center of Oasis Agricultural Resources and Environment in Southern Xinjiang, Tarim University, Alar, 843300, China; Research Institute of Farmland Water Conservancy and Soil Fertilizer, Xinjiang Academy of Agricultural Reclamation Sciences, Shihezi, 832000, China; Institute of Forestry and Horticulture, Xinjiang Academy of Agricultural Sciences, Shihezi, 832000, China; Institute of Mechanical Equipment, Xinjiang Academy of Agricultural Sciences, Shihezi, 832000, China

**Keywords:** water–fertilizer coupling, arid region, fruit quality, pear

## Abstract

Considering the pear in the arid region as the research object, single-factor testing and water–fertilizer coupling testing were conducted. The response of pear tree growth to water, nitrogen, and phosphorus was explored and provided a theoretical basis for efficient water and fertilizer management. Among them, the single-factor test set water, nitrogen, and phosphorus as the three factors, and five levels were set. Screening out W3, W4, N3, N4, P3, and P4 promoted plant nutrient uptake and fruit quality. Eight treatments were set up in the water and fertilizer coupling test: Treatment 1 (T1, W3N3P3), Treatment 2 (T2, W3N3P4), Treatment 3 (T3, W3N4P3), Treatment 4 (T4, W3N4P4), Treatment 5 (T5, W4N3P3), Treatment 6 (T6, W4N3P4), Treatment 7 (T7, W4N4P3), and Treatment 8 (T8, W4N4P4). The results showed that the leaf area index of the T1, T2, T3, and T4 treatments was significantly higher than that of the other treatments at maturity. The yield, single fruit weight, and primary fruit rate were the highest under T3 treatment. The gray correlation degree analysis of fruit quality showed that the T3 treatment had the highest degree of correlation and ranking of each fruit quality index, indicating that the T3 treatment had the highest fruit quality. The yield model showed that irrigation with 6510.06 m^3^ hm^−2^, nitrogen fertilizer with 337.5 kg N hm^−2^, and phosphate fertilizer with 262.5 kg P hm^−2^ had the best yield. A detailed investigation of pear tree growth and fruit quality showed that the T3 treatment had the best fruit growth and development performance, and the pear fruit quality was the best.

## 1 Introduction

Korla fragrant pear is one of the most famous fruits in Xinjiang. It belongs to the Rosaceae pear subfamily pear plant [1] and is a cross between an Asian pear and a European pear. It is a small fruit, with the weight of a single fruit being about 90–150 g, and it has the characteristics of thin skin and fine meat, crisp and refreshing [2]. Pear trees have strict requirements for temperature and light conditions [3]. The soil in the Xinjiang region is deep and fertile, the light is sufficient, the temperature difference between day and night is large, the precipitation is low, and the evaporation is high, which can greatly reduce the occurrence of pear tree diseases and insect pests. Therefore, it is highly suitable for pear tree planting. Water and fertilizer management is the basis of pear yield. With the long-term development of Xinjiang’s forest and fruit industry, water and fertilizer management measures have been continuously optimized, and the yield has also increased. As of 2017, Xinjiang’s pear tree planting area reached 62,800 hm^2^, with an output of 1.231 million tons, making it one of the major pear fruit-producing areas in China [4].

Strategic water and fertilizer management are the bases for the high yield and quality of pear trees [5]. However, improper cultivation management can occur, resulting in low fruit quality rates, high inputs, and low outputs. In recent years, with the development of science and technology, promoting the use of water with fertilizer, promoting the use of fertilizer with water, and coupling water and fertilizer are key technologies to improve water and fertilizer use efficiency and reduce environmental pollution [6]. Strategic water and fertilizer management can play an important role in improving the quality and yield of fruit trees. It can provide a favorable environment for the growth and development of fruit trees and enable them to achieve the virtuous cycle of high quality and high yield [7]. However, during the production process, fruit farmers generally implement the management protocols of large quantities of water and fertilizer. Problems from excessive water and fertilizer inputs and unsustainable distribution of water and fertilizer are prominent. Chai et al. [8] investigated the planting of fragrant pear in 15 households and showed that the organic fertilizer of fragrant pear was 15,000 m^3^ hm^−2^, and the input of chemical fertilizer was 2,550 m^3^ hm^−2^. Liu et al. [9] found that the irrigation volume of pear in the arid region reached 14,400 m^3^ hm^−2^, which was substantially higher than the recommended irrigation volume. Unsustainable water and fertilizer management, soil compaction, and permeability can decrease, resulting in a tree nutritional imbalance that affects fruit quality. Therefore, it is necessary to conduct research on the water and fertilizer regulation of pear in arid regions, which has important theoretical and practical importance in maintaining the sustainable development of the pear industry.

Xinjiang is located inland, with a dry climate and frequent water shortages. Meanwhile, Xinjiang soil is rich in potassium because of the influence of the parent material and climate. Water, nitrogen, and phosphorus management have become the focus of fruit tree water and fertilizer management in Xinjiang. In this study, the effects of water, nitrogen, and phosphorus on the nutrient absorption and fruit quality of the pears were investigated through factor tests to determine the appropriate amounts of water, nitrogen, and phosphorus. A coupling test of water, nitrogen, and phosphorus was used to examine the effects of water and fertilizer coupling on the growth, fruit yield, and fruit quality of the pears to provide theoretical and technical support for strategic water and fertilizer management of the pears.

## 2 Materials and methods

### 2.1 Summary of experimental sites

The study was conducted at the 14th Company of the 9th Regiment of Alar City of the 1st Agricultural Division of Xinjiang Production and Construction Corps. The study area is a continental arid desert climate with average annual sunshine of 2556.3–2991.8 h, average annual precipitation of 40.1–82.5 mm, and average annual evaporation of 1876.6–2558.9 mm. The physical and chemical properties of the test sites are listed in Table 1. The soil conductivity is 104.64 μs cm^−1^, pH is 8.45, organic matter is 5.10 g kg^−1^, the salt content is 0.45 g kg^−1^, and alkali-hydrolytic nitrogen, available phosphorus, and available potassium are 6.51, 17.31, and 70.38 mg kg^−1^, respectively.

**Table 1:** Fertility of the test site

	**S**oil depth			
Index	0–20 cm	20–40 cm	40–60 cm	60–80 cm
Soil bulk density (g cm^−3^)	1.60	1.56	1.43	1.38
pH	8.37	8.45	8.48	8.51
Electric conductivity (μs cm^−1^)	119	117	92.05	90.5
Organic matter (g kg^−1^)	8.16	6.44	4.36	1.43
Salinity (g kg^−1^)	0.61	0.68	0.71	0.74
Alkali-hydrolyzed nitrogen (mg kg^−1^)	12.75	8.05	3.85	1.4
Rapidly available phosphorus (mg kg^−1^)	27.29	26.86	10.50	4.57
Rapidly available potassium (mg kg^−1^)	93	81.5	70	37

### 2.2 Test materials and design

The experimental subjects were grafted 5-year-old pear trees with a row spacing of 1.5 m × 4 m. The soil type was sandy loam, and drip irrigation was used. The phenomenon of excessive fertilizer application is common in orchards in southern Xinjiang, so the coupling test of water and fertilizer based on the conventional fertilizer application of farmers cannot accurately represent the reference of water and fertilizer suitable for high yield and high quality of orchards in this region. Therefore, based on the physical and chemical properties of the soil in the area, the results of previous studies, and the local fertilization situation, the first-year single-factor test dosage was determined to determine the appropriate water, nitrogen, and phosphorus inputs. In the second year, the water–fertilizer coupling test was carried out based on the results of the first-year test to determine the high-yield and high-quality water–fertilizer coupling model of pear trees in this area.

A single-factor experiment was conducted using a completely random design. A single-factor experiment was set up with three factors and five levels for each (Table 2). Among them, N3 and P3 were the experimental routine fertilizer rates; N1 and P1 were the ultra-low fertilizer rate and 0.5 times the conventional fertilizer rate; N2 and P2 were the low fertilizer rate and 0.75 times the conventional fertilizer rate; N4 and P4 were the high fertilizer rate and 1.25 times the conventional fertilizer rate; and N5 and P5 were the ultra-high fertilizer rate and the conventional fertilizer rate 1.5 times, for a total of 15 processes. Each treatment was repeated three times, and 45 plots were selected. Ten pear trees with similar growth rates were selected from each plot. The water and fertilizer coupling experiment was designed as a three-factor, two-level, completely randomized block design with a total of eight treatments. Each treatment was repeated three times, and 24 plots were divided (Table 3).

**Table 2:** Single-factor experimental design

ID	Experimental factor	Factor level	Processing code
1	Irrigation quantity during the growth period (m^3^ hm^−2^)	5,460	W1
2		5,880	W2
3		6,300	W3
4		6,720	W4
5		7,140	W5
6	Nitrogen fertilizer application rate (kg N hm^−2^)	150	N1
7		225	N2
8		300	N3
9		375	N4
10		450	N5
11	Amount of phosphate fertilizer applied (kg P_2_O_5_ hm^−2^)	75	P1
12		150	P2
13		225	P3
14		300	P4
15		375	P5

**Table 3:** Design of water and fertilizer coupling test

ID	Processing code	Irrigation quantity during the growth period	Nitrogen fertilizer application rate	Amount of phosphate fertilizer applied
		(m^3^ hm^−2^)	(kg N hm^−2^)	(kg P_2_O_5_ hm^−2^)
1	T1	6,300	300	225
2	T2	6,300	300	300
3	T3	6,300	375	225
4	T4	6,300	375	300
5	T5	6,720	300	225
6	T6	6,720	300	300
7	T7	6,720	375	225
8	T8	6,720	375	300

### 2.3 Index determination method

#### 2.3.1 Determination of the plant growth index

For determination of the leaf area index, three non-adjacent fruit trees were selected from each plot, and a canopy analysis instrument (LP-80) was used for five canopy measurements at the flowering (mid–late March to early April), young fruit development (mid–late April to early May), early fruit expansion (mid-May to early June), late fruit expansion (mid-June to late July), and maturity (early August) stages. For assessment of the root growth index, three non-adjacent fruit trees were selected from each plot, and root samples were collected before and after fruit harvesting. LA-S was used for scanning, and the WinRHIZO system was used to measure the root density and root surface area. For determination of the yield, at the fruit maturity stage, 3 non-adjacent fruit trees were selected in each plot, all the fruits were picked and weighed, and 20 fruits were randomly selected to determine the single fruit weight.

#### 2.3.2 Leaf collection and nutrient determination

Four pear trees with similar growth were randomly selected from each plot, and one new branch and one biennial branch were selected from the east, west, south, and north directions, respectively. New branches were collected at the early fruit development stage (mid–late April to early May), early fruit expansion stage (mid-May to early June), late fruit expansion stage (mid-June to late July), and maturity stage (early August). Fifteen leaves were collected from each raw and second-year branch, labeled, and brought back to the laboratory for the determination of total nitrogen and total phosphorus content. The leaves were boiled in H_2_SO_4_–H_2_O_2_, the total nitrogen content was determined using the Kay nitrogen determination method, and the total phosphorus content was determined using the vanadium–molybdenum yellow colorimetric method.

#### 2.3.3 Determination of the fruit quality index

For the determination of the fruit shape index, during the young fruit growth stage, four non-adjacent fruit trees were selected from each plot, and the fruit trees were divided into upper and lower layers. One disease-free fruit with a uniform shape was selected from each side of the selected fruit trees. Eight healthy young fruits were randomly selected from each plot, and the longitudinal and transverse diameters of the fruits were measured every 15 days using digital Vernier calipers. The pear fruit shape index was calculated as follows:

\[\text{Fruit shape index = Fruit longitudinal diameter/Fruit transverse diameter}.]\]

For the determination of pear fruit quality, at the fruit ripening stage, 30 fresh fruits were randomly selected from each plot to determine fruit quality indices. Fruit hardness, stone cell content, soluble solids, soluble sugar content, titratable acid content, vitamin C content, total phenol content, and flavonoid content were determined. Fruit hardness was measured using a fruit hardness tester. The fruit stone cell content was determined using a freezing method. Fruit soluble solids (handheld saccharometer), soluble sugars (concentrated sulfate–anthranone colorimetric method), titratable acids (ethanol extraction–lye titration method), vitamin C (fluorescence colorimetric method), total phenols, and flavonoids (spectrophotometric–colorimetric method) were examined.

### 2.4 Data processing and analysis

Excel 2016 was used to organize, analyze, and calculate the test data. DPS 25 software was used for variance and significance analyses (*p* < 0.05). The least-squares method was used to draw a nonlinear regression fitting curve to fit the fruit growth trend under different treatments, in which time was the independent variable and fruit shape index was the dependent variable. The fruit quality index was analyzed using the gray correlation degree. The correlation coefficient represented the correlation degree value of the corresponding dimension between the subsequence and the parent sequence, and the larger the number, the stronger the generation correlation. The correlation coefficients under different treatments were compared to obtain the water–fertilizer coupling treatment with a higher degree of correlation and better fruit quality. Origin 2018 software was used to create the relevant charts for the test data.

## 3 Results

### 3.1 Effects of the amount of water and fertilizer on leaf nutrient change dynamics in pears

#### 3.1.1 Effects of water and fertilizer dosage on total nitrogen in pear leaves

**Figure 1:**
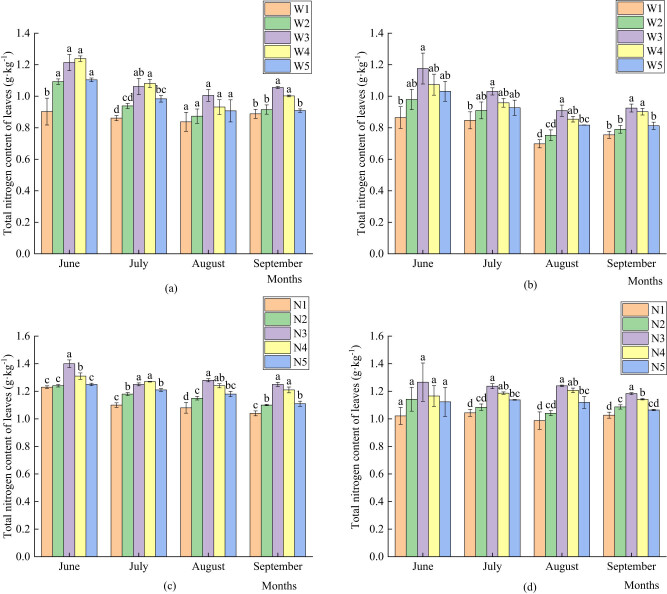
Effects of different amounts of water and fertilizer on the total nitrogen content in leaves. (a) Total nitrogen content of leaves of current-year branches under different irrigation levels; (b) total nitrogen content of leaves of perennial branches under different irrigation amounts; (c) total nitrogen content in leaves of current-year branches under different nitrogen application rates; and (d) total nitrogen content of leaves of perennial branches under different nitrogen application rates.

As shown in Figure 1, with the progress of the growth period, the leaves of both the current and perennial branches showed a trend of first decreasing and then slowly increasing. The main reason for this was that pear trees were in the vegetative growth stage in the early stages and stored substantial amounts of nutrients. However, as the growth period progressed, pear trees shifted from vegetative to reproductive growth, and the nutrients stored in the pear trees migrated to the developing fruits. Therefore, the residual nutrients in the leaves began to decline. By September, the fruits were mature, nutrients in the tree had reached saturation, and parts began to accumulate. The amount of irrigation significantly affected the total nitrogen content of the pear leaves in the arid region, and the total nitrogen content of leaves of annual branches and perennial branches treated with W3 and W4 was the highest. In September, the total nitrogen content of the leaves of annual and perennial branches treated with W3 and W4 was significantly higher than that of the other treatments. The nitrogen fertilizer application rate significantly affected the total nitrogen content of the pear leaves in the arid region, and the total nitrogen content of the pear leaves in the arid region treated with N3 and N4 was relatively high. In June, July, and September, the total leaf nitrogen content of the annual branches treated with N3 and N4 was significantly higher than that of the other treatments. In July, August, and September, the total nitrogen content of the perennial branches treated with N3 was significantly higher than that of the other treatments.

#### 3.1.2 Influence of water and fertilizer dosage on total phosphorus of pear leaves

**Figure 2:**
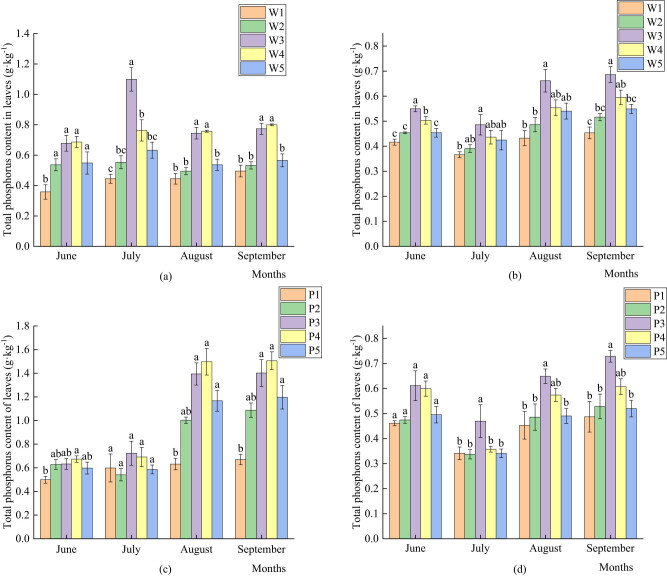
Effects of different amounts of water and fertilizer on the total phosphorus content in leaves. (a) Total nitrogen content of leaves of current-year branches under different irrigation levels; (b) total nitrogen content of leaves of perennial branches under different irrigation amounts; (c) total nitrogen content in leaves of current-year branches under different nitrogen application rates; and (d) total nitrogen content of leaves of perennial branches under different nitrogen application rates.

As shown in Figure 2, the total phosphorus content of the leaves of perennial branches was lower than that of the leaves of current branches. The reason for this phenomenon is that June and July are the periods of more vigorous fruit development, their respiration is significant, and they consume higher quantities of phosphorus compared with the mature stage. The leaves of the current branches have stronger activity than the leaves of the perennial branches. Therefore, the leaves of the current branches have a stronger accumulation capacity for phosphorus. In August and September, the growth of the tree body was slow, respiration was weakened, and the leaf phosphorus consumption was lower than that of the previous period. Irrigation amount significantly affected the total phosphorus content in the leaves of pear in the arid region, and the W3 and W4 treatments had the highest total phosphorus content in the leaves of annual and perennial branches. The amount of P fertilizer significantly affected the total P content in the pear leaves. In July, August, and September, the total P content of P3-treated perennial branches was significantly higher than that of the other treatments.

### 3.2 Influence of water and fertilizer dosage on the fruit quality of pears

#### 3.2.1 Influence of irrigation amount on the fruit quality of pears

Table 4 shows that the amount of irrigation significantly affected the fruit quality of pears. Among them, the contents of Vc, soluble solids, and soluble sugar were in the following order: W3 > W4 > W5 > W1 > W2. The Vc content of fruits treated with W3 and W4 significantly increased by 5.1–15.8% compared with other treatments. The soluble solids content of fruits treated with W3 and W4 significantly increased by 5.8–11.2% compared with other treatments. The soluble content of fruits treated with W3 and W4 significantly increased by 5.8–11.2% compared with other treatments. Compared with other treatments, the sugar content significantly increased by 6.2–22.9%, and the titratable acid content of fruits treated with W3 and W4 significantly decreased by 2.1–26.3%. However, the stone cell content of fruits treated with W3 and W4 significantly increased compared with other treatments. In conclusion, the fruit quality of pears with W3 and W4 treatments was the highest.

**Table 4:** Effects of the amount of irrigation on the pear fruit quality

Test treatment	Vc (mg/100 g)	Soluble solid content	Stone cell content (%)	Soluble sugar (%)	Titratable acid (%)
W1	8.71 ± 0.13d	10.43 ± 0.25c	2.50 ± .033d	16.17 ± 1.85c	1.24 ± 0.02a
W2	9.31 ± 0.07c	10.78 ± 0.34b	3.20 ± 0.04c	16.97 ± 0.76c	1.17 ± 0.06b
W3	10.10 ± 0.13a	11.61 ± 0.26a	3.66 ± 0.55ab	19.87 ± 0.11a	0.95 ± 0.03d
W4	9.82 ± 0.02b	11.56 ± 0.24a	3.82 ± 0.11a	18.71 ± 0.88ab	1.00 ± 0.03c
W5	9.36 ± 0.06c	10.92 ± 0.41b	3.18 ± 0.38bc	17.62 ± 0.64bc	1.02 ± 0.03c

Note: Lowercase letters a, b, c, d indicate the difference between treatments (*P* < 0.05).

#### 3.2.2 Effects of the amount of nitrogen application on the fruit quality of pears

As shown in Table 5, the amount of nitrogen applied significantly affected the fruit quality of pears. Among them, the contents of Vc, soluble solid matter, soluble sugar, and stone cells were N3 > N4 > N5 > N1 > N2. The Vc content of fruits treated with N3 and N4 significantly increased by 1.9–20.8% compared with other treatments. The soluble sugar content of fruits treated with N3 and N4 significantly increased by 9.9–41.9% compared with other treatments, and the fruit treated with N3 and N4 significantly increased by 9.9–41.9%. The content of solid stone cells significantly increased by 4.5–37.9% compared with other treatments, and the titratable acid content of fruits treated with N3 and N4 significantly decreased by 2.1–25.4% compared with other treatments. In conclusion, the fruit quality of pears treated with N3 and N4 was the highest.

**Table 5:** Effects of nitrogen application on pear fruit quality

Test treatment	Vc (mg/100 g)	Soluble solid content	Stone cell content (%)	Soluble sugar (%)	Titratable acid (%)
N1	9.20 ± 0.15e	10.94 ± 0.32c	2.53 ± 0.46c	14.44 ± 2.01c	1.14 ± 0.09a
N2	9.71 ± 0.11d	11.38 ± 0.39b	2.81 ± 0.26bc	15.09 ± 1.05c	1.03 ± 0.04b
N3	11.11 ± 0.06a	12.27 ± 0.42a	3.49 ± 0.03a	20.49 ± 2.84a	0.85 ± 0.02c
N4	10.31 ± 0.13b	12.04 ± 0.09a	3.25 ± 0.22a	20.01 ± 2.86ab	0.95 ± 0.02b
N5	10.12 ± 0.04c	11.42 ± 0.50b	3.11 ± 0.07ab	18.20 ± 2.14b	0.97 ± 0.03b

Note: Lowercase letters a, b, c, d indicate the difference between treatments (*P* < 0.05).

#### 3.2.3 Effects of the amount of phosphorus application on the fruit quality of pears

As shown in Table 6, the amount of phosphorus applied significantly affected the fruit quality of pears. Among them, the contents of Vc, soluble sugar, and stone cells were in the following order: P3 > P4 > P5 > P1 > P2. The Vc content of fruits treated with P3 and P4 significantly increased by 6.3–21% compared with other treatments. The soluble solid content of fruits treated with P3 and P4 significantly increased by 1.3–9.8% compared with other treatments, and the soluble sugar content of fruits treated with P3 and P4 significantly increased by 1.3–9.8%. Compared with other treatments, the fruit stone cell content of P3 and P4 treatments significantly increased by 6.1–61.2%, and the titratable acid content of fruits of P3 and P4 treatments significantly decreased by 14.8–28.5%. In conclusion, the fruit quality of pears with the P3 and P4 treatments was the highest.

**Table 6:** Effect of the amount of phosphorus application on the pear fruit quality

Test treatment	Vc (mg/100 g)	Soluble solid content	Stone cell content (%)	Soluble sugar (%)	Titratable acid (%)
P1	8.25 ± 0.04d	10.37 ± 0.28c	2.72 ± 0.32d	12.01 ± 3.01c	1.23 ± 0.05a
P2	8.70 ± 0.08c	10.88 ± 0.35b	2.93 ± 0.34cd	13.28 ± 2.47c	1.19 ± 0.06a
P3	9.98 ± 0.06a	11.39 ± 0.32a	3.56 ± 0.32a	19.36 ± 1.11a	0.88 ± 0.06c
P4	9.92 ± 0.12a	11.02 ± 0.21b	3.40 ± 0.07ab	16.94 ± 1.51b	0.92 ± 0.09c
P5	9.33 ± 0.06b	10.59 ± 0.30c	3.12 ± 0.19bc	15.96 ± 1.25b	1.08 ± 0.02b

Note: Lowercase letters a, b, c, d indicate the difference between treatments (*P* < 0.05).

Overall, the W3, W4, N3, N4, P3, and P4 treatments showed the best leaf nutrient use and fruit quality. Therefore, the W3 (6,300 m^3^ hm^−2^), W4 (6,720 m^3^ hm^−2^), N3 (300 kg N hm^−2^), N4 (375 kg N hm^−2^), P3 (225 kg P_2_O_5_ hm^−2^), and P4 (300 kg P_2_O_5_ hm^−2^) treatments were applied as the fertilization and irrigation levels used in the coupling test.

### 3.3 Influence of water and fertilizer coupling on the pear growth

#### 3.3.1 Effects of water and fertilizer coupling on the pear root density and root surface area

As shown in Table 7, the root density and root surface area of the 40–60 cm soil layer were higher than those of the 20–40 cm soil layer and those of the 0–20 cm soil layers. This indicated that the roots were mainly concentrated in the 40–60 cm soil layer. The coupling of water, nitrogen, and phosphorus had no significant effect on the 0–60 cm root density of the pear in the arid region. The coupling of water, nitrogen, and phosphorus significantly affected the root surface area of the 0–60 cm soil layer. At the early stages of flowering, the root surface area of 0–20 cm soil layer was in the following order: T6 > T1 > T5 > T7 > T3 > T2 > T4 > T8, and that of 20–40 cm soil layer was in the following order: T1 > T2 > T6 > T7 > T3 > T5 > T4 > T8. In the 40–60 cm layer, the root surface area was in the following order: T1 > T4 > T7 > T3 > T6 > T2 > T8 > T5.

**Table 7:** Effects of water and fertilizer coupling on the root density and root surface area of pears before flowering

Test treatment	0–20 root density (cm cm^−3^)	20–40 root density (cm cm^−3^)	40–60 root density (cm cm^−3^)	0–20 root surface area (cm^2^)	20–40 root surface area (cm^2^)	40–60 root surface area (cm^2^)
T1	0.06 ± 0.015a	0.09 ± 0.006a	0.16 ± 0.01a	8.64 ± 1.93ab	17.78 ± 2.23a	22.4 ± 1.22a
T2	0.04 ± 0.012a	0.11 ± 0.006a	0.15 ± 0.01a	5.49 ± 0.51c	13.78 ± 0.99bc	17.52 ± 1.32b
T3	0.05 ± 0.006a	0.10 ± 0.006a	0.15 ± 0.01a	6.22 ± 1.66c	12.02 ± 2.05b	18.66 ± 1.11b
T4	0.04 ± 0.01a	0.07 ± 0.01a	0.12 ± 0.01a	5.35 ± 1.12c	11.03 ± 0.8bc	21.92 ± 1.42a
T5	0.05 ± 0.006a	0.07 ± 0.006a	0.13 ± 0.01a	6.77 ± 0.75bc	12.01 ± 1.63b	17.29 ± 1.3b
T6	0.05 ± 0.006a	0.10 ± 0.01a	0.14 ± 0.03a	9.87 ± 1.09a	13.37 ± 0.64b	18.23 ± 1.44b
T7	0.04 ± 0.006a	0.09 ± 0.006a	0.14 ± 0.01a	6.56 ± 0.94bc	12.76 ± 2.32b	21.62 ± 1.06a
T8	0.04 ± 0.006a	0.07 ± 0.006a	0.11 ± 0.01a	5.28 ± 2.17c	9.05 ± 1.7c	17.44 ± 0.78b

Note: Lowercase letters a, b, c, d indicate the difference between treatments (*P* < 0.05).

As shown in Table 8, the root change trend was consistent with that of the early flowering period, indicating that the root system was mainly concentrated at soil depths of 40–60 mm. After fruit harvest, the root surface area of the 0–20 cm soil layer was in the following order: T1 > T5 > T6 > T7 > T2 > T3 > T8 > T4. The root surface area of the 20–40 cm soil layer was in the following order: T1 > T5 > T7 > T4 > T6 > T3 > T2 > T8. The root surface area of the 40–60 cm soil layer was in the following order: T7 > T1 > T4 > T8 > T3 > T2 > T5 > T6.

**Table 8:** Effects of water and fertilizer coupling on the root density and root surface area of pears after harvest

Test treatment	0–20 root density (cm cm^−3^)	20–40 root density (cm cm^−3^)	40–60 root density (cm cm^−3^)	0–20 root surface area (cm^2^)	20–40 root surface area (cm^2^)	40–60 root surface area (cm^2^)
T1	0.06 ± 0.01a	0.11 ± 0.01a	0.21 ± 0.01a	12.88 ± 0.65a	19.96 ± 0.62a	28.38 ± 1.36a
T2	0.06 ± 0.02a	0.12 ± 0.012a	0.17 ± 0.006a	9.91 ± 0.93bc	14.40 ± 2.15bc	23.37 ± 1.8c
T3	0.05 ± 0.01a	0.12 ± 0.006a	0.19 ± 0.01a	9.66 ± 2.3bc	15.07 ± 1.09bc	24.11 ± 1.56bc
T4	0.06 ± 0.01a	0.12 ± 0.01a	0.16 ± 0.01a	7.79 ± 1.61c	16.58 ± 3.56b	26.41 ± 0.9ab
T5	0.07 ± 0.01a	0.12 ± 0.012a	0.18 ± 0.006a	11.14 ± 1.09ab	16.83 ± 3.52b	20.31 ± 0.89d
T6	0.06 ± 0.01a	0.11 ± 0.006a	0.16 ± 0.006a	11.11 ± 1.69ab	16.36 ± 1.56b	19.37 ± 0.33d
T7	0.07 ± 0.01a	0.13 ± 0.012a	0.16 ± 0.006a	10.38 ± 1.81abc	16.69 ± 0.95b	28.61 ± 1.53a
T8	0.06 ± 0.01a	0.13 ± 0.006a	0.15 ± 0.01a	8.36 ± 2.17bc	12.31 ± 0.94c	24.25 ± 1.92bc

Note: T1 (W3N3P3), T2 (W3N3P4), T3 (W3N4P3), T4 (W3N4P4), T5 (W4N3P3), T6 (W4N3P4), T7 (W4N4P3), and T8 (W4N4P4). Lowercase letters a, b, c, d indicate the difference between treatments (*P* < 0.05).

#### 3.3.2 Influence of water and fertilizer coupling on the change in the leaf area index of pears

**Figure 3:**
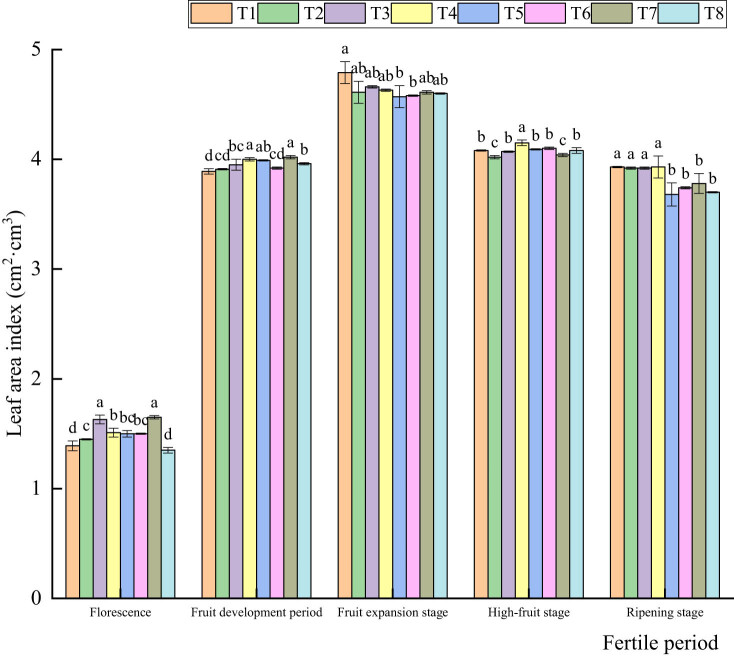
Effects of different water and fertilizer coupling treatments on the leaf area index. Note: T1 (W3N3P3), T2 (W3N3P4), T3 (W3N4P3), T4 (W3N4P4), T5 (W4N3P3), T6 (W4N3P4), T7 (W4N4P3), T8 (W4N4P4).

As shown in Figure 3, the results showed that the overall change trend in the leaf area index showed first an increase and then a slight decrease. At the flowering and early fruit development stages, the leaf area index under the T3 and T7 treatments was significantly higher than that under the other treatments. The leaf area index increased rapidly during the fruit expansion stage. At the strong fruit stage, the LAI began to decrease with dead branches and leaves. The change in the leaf area index under the T1 treatment was the largest, decreasing by 0.71 cm^2^ cm^3^. At maturity, the leaf area index under the T5 treatment showed the largest change, and the leaf area index under the T1–T4 treatments was significantly higher than that under the other treatments. In general, the leaf area index reached its maximum during the fruit expansion stage, and the leaf area index began to decline during the strong fruit stage.

#### 3.3.3 Influence of water and fertilizer coupling on the fruit shape index of pears

**Figure 4:**
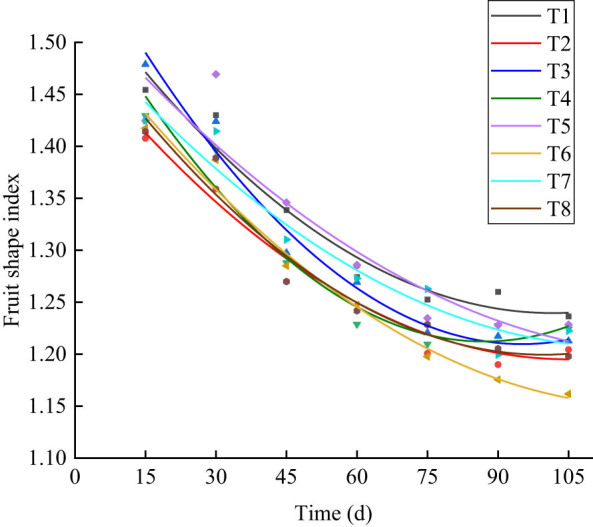
Fitted growth curves of the fruit shape index under different water and fertilizer coupling treatments. Note: T1 (W3N3P3), T2 (W3N3P4), T3 (W3N4P3), T4 (W3N4P4), T5 (W4N3P3), T6 (W4N3P4), T7 (W4N4P3), and T8 (W4N4P4).

As shown in Figure 4, the least-squares method was used to fit the fruit shape index curve for the different measurement days. Eight processing, according to the results of regression fitting curve determination coefficient (*R* ^2^), is shown as T3 (0.541) > T6 (0.408) > T4 (0.407) > T5 (0.375) > T2 (0.340) > T7 has (0.322) > T1 (0.317) > T8 (0.294). From the goodness of fit, the growth trend of the fruit shape under the T3 treatment was closest to the fitting growth curve, and *R* ^2^ was closest to 1, which was consistent with the growth law.

#### 3.3.4 Influence of water and fertilizer coupling on the yield of pears

As shown in Table 9, there was a significant difference in fruit-per-fruit weight between different water–fertilizer coupling treatments, with fruit-per-fruit weight ranging from 137.61 to 114.39 g. Among these, the T3 treatment had the largest fruit-per-fruit weight (137.61 g). The fruit-per-fruit weight between different treatments was in the following order: T3 > T1 > T5 > T8 > T4 > T3 > T7. The total fruit yield differed under the different water and fertilizer combined treatments, and the total fruit yield was in the following order: T3 > T6 > T2 > T7 > T1 > T5 > T4 > T8. The primary fruit production rate for each treatment was in the following order: T3 > T7 > T2 > T6 > T8 > T1 > T5 > T4. The primary fruit rate in the T3 treatment was the highest (78.14%), and the fruit rate in the T4 treatment was the lowest (64.44%). The T3 treatment had the highest fruit yield, the largest single fruit weight, and the highest primary fruit rate.

**Table 9:** Changes in the fruit yield and single fruit weight under different water and fertilizer coupling treatments

Test treatment	Weight of single fruit (g)	Yield (kg hm^−2^)	Primary fruit rate (%)	Secondary fruit rate (%)	Tertiary fruit rate (%)
T1	130.28 ± 11.17ab	4388.89c	67.09d	18.38b	14.53a
T2	122.50 ± 12.01ab	4463.89bc	73.62bc	12.76d	13.63ab
T3	137.61 ± 6.115a	4916.67a	78.14a	9.72e	12.15bc
T4	125.40 ± 3.383ab	4194.44d	64.44e	21.85a	13.71ab
T5	129.90 ± 5.661ab	4258.33d	64.83e	20.73a	14.45a
T6	115.31 ± 7.566b	4533.33b	72.43c	16.30c	11.27c
T7	114.39 ± 6.496b	4447.22bc	74.29b	16.21c	9.49d
T8	126.38 ± 14.97ab	4091.67d	72.03c	18.86b	9.10d

Note: T1 (W3N3P3), T2 (W3N3P4), T3 (W3N4P3), T4 (W3N4P4), T5 (W4N3P3), T6 (W4N3P4), T7 (W4N4P3), and T8 (W4N4P4). Lowercase letters a, b, c, d indicate the difference between treatments (*P* < 0.05).

### 3.4 Effects of water and fertilizer coupling on the fruit quality of pears

The results of the gray correlation degree analysis showed that the stone cell content, fruit hardness, Vc content, soluble sugar content, total phenol content, and titratable acid content of the fruit had a strong correlation with the fruit quality of the pear in the arid region and were the key indicators affecting the fruit quality. The results of the gray relational degree analysis showed that the quality of the pear in the arid region was in the following order: T3 > T1 > T7 > T5 > T2 > T8 > T6 > T4. The fruit quality of the pear in the arid region treated with treatment T3 was the highest (Table 10).

**Table 10:** Gray relational degree analysis

Correlation coefficient results								
Test treatment	T1	T2	T3	T4	T5	T6	T7	T8
Stone cell content	0.9748	0.9532	0.9941	0.9524	0.9511	0.9349	1.0000	0.9653
Hardness	0.8212	0.8078	0.8048	0.8043	0.8063	0.7980	0.8049	0.8143
Soluble solid	0.3449	0.3622	0.3505	0.3659	0.3593	0.3662	0.3714	0.3504
Soluble sugar	0.6353	0.5874	0.6395	0.6120	0.6132	0.5997	0.5561	0.5871
Titratable acid	0.5391	0.5332	0.5414	0.5314	0.5448	0.5260	0.5384	0.5385
Vc	0.7382	0.7461	0.7433	0.7408	0.7487	0.7787	0.7449	0.7081
Total phenol	0.5851	0.5640	0.5714	0.5285	0.5622	0.5428	0.5951	0.5869
Flavonoid	0.3757	0.3752	0.3762	0.3750	0.3755	0.3753	0.3757	0.3759
Sort	2	5	1	8	4	7	3	6

Note: T1 (W3N3P3), T2 (W3N3P4), T3 (W3N4P3), T4 (W3N4P4), T5 (W4N3P3), T6 (W4N3P4), T7 (W4N4P3), and T8 (W4N4P4).

### 3.5 The establishment of the yield model

The regression model of yield, nitrogen, phosphorus, and water consumption was obtained by using ternary quadratic regression fitting between yield and nitrogen, phosphorus, and water consumption among treatments obtained in the experiment:

(1) \[Y\text{=}-\text{12305}{\text{.8 \times N}}^{\text{2}}+\text{17933}{\text{.29 \times P}}^{\text{2}}+\text{4}{\text{.108 \times W}}^{\text{2}}\hspace{2em}+\text{8306532 \times N}-\text{9414994 \times P}-\text{53486}\text{.3 \times W}\hspace{2em}-\text{0}\text{.127 \times N \times P}-\text{0}\text{.008 \times N \times W + 0}\text{.009 \times P \times W}\text{.}]\]

By the significance test of equation (1), *R* ^2^ = 0.85, indicating a good fit between the predicted value and the true value. Among them, the coefficient of the primary term is 8306532, −9414994, and −53486.3, respectively, indicating that the influence of each factor on the yield is N > W > P, and the interaction effect coefficient is −0.127, −0.008, and 0.009, indicating that the influence of the interaction effect of each factor on the yield is P × W > N × W > N × P.

In order to explore the optimal amount of water and fertilizer under these conditions, by differentiating equation (1) by partial differentiation, the following equations can be obtained:

(2) \[\text{d}Y/\text{d}{C}_{\text{N}}=-24611.6\text{}\times \text{}{C}_{\text{N}}-0.127\times \text{}{C}_{\text{P}}-0.008{C}_{\text{W}}+8306532,]\]

(3) \[\text{d}Y/\text{d}{C}_{\text{P}}=-0.127\hspace{.5em}\times {C}_{\text{N}}+35866.58\times {C}_{\text{P}}+0.009\times {C}_{\text{W}}-9414994,]\]

(4) \[\text{d}Y/\text{d}{C}_{\text{W}}=-0.008\text{}\hspace{.25em}\times \text{}{C}_{\text{N}}+\text{}0.009\text{}\times \text{}{C}_{\text{P}}+\text{}8.216\text{}\times \text{}{C}_{\text{W}}-53486.3.]\]

Having d*Y*/d*C*_N_ = 0, d*Y*/d*C*_P_ = 0, and d*Y*/d*C*_W_ = 0, the optimal solution of equation (1) *C*_N_ = 337.5, *C*_P_ = 262.5, and *C*_W_ = 6510.06 is obtained. Therefore, it can be concluded that 6510.06 m^3^ hm^−2^ irrigation, 337.5 kg N hm^−2^ nitrogen, and 262.5 kg P hm^−2^ phosphorus application are the best water and fertilizer application in this area during the growth period.

## 4 Discussion

### 4.1 Effects of water, nitrogen, and phosphorus on the pear growth in the arid region and fruit quality

Water is the most important factor in plant growth. In the case of drought and water shortage, the soil cannot provide water for the fruit trees in time. This weakens the plant’s photosynthetic and metabolic capacity, thereby reducing the accumulation of organic matter in plants. When soil moisture is sufficient, fruit trees can obtain a lot of supplementary water from the soil environment. Instead, it promotes the synthesis of organic matter in plants. The nutrients for fruit growth come from the accumulation of nutrients in the tree, and leaf nutrients are the storage of nutrients required for fruit growth. Therefore, the level of leaf nutrients depends on the nutrient absorption and utilization of the tree, which determines the amount of nutrients supplied to the fruit. In this study, under the drip irrigation mode, the total nitrogen and total phosphorus contents in Korla pear leaves were the highest when the irrigation amount was 6,300–6,720 m^3^ hm^−2^, and the fruit quality of Korla pears was higher. In line with the results of this study, Liu et al. [10] found that appropriate irrigation promoted the nutrient use efficiency of mangoes. Chen et al. [11] found that appropriate irrigation reduced reactive oxygen species content in the calyx tube of pears and promoted the quality of pears. This is mainly because suitable irrigation promoted the root growth of the pear, improved the water conductivity of the pear in the arid region, improved the transport efficiency of nutrients absorbed by the root system to the aboveground parts, and improved the fruit quality of the pear using the accumulation of nutrients in leaves [12].

Nitrogen and phosphorus are essential nutrients for plant growth and play irreplaceable roles in the growth and development of fruit trees [13,14,15]. The synthesis of proteins and chlorophyll in fruit trees requires nitrogen, and the lack of nitrogen affects the physiological growth, fruit yield, and fruit quality. Similarly, research results on phosphorus have found that insufficient phosphorus supply severely affects the absorption of nitrogen by plants. Meanwhile, excessive phosphorus application inhibits the normal growth of plants and reduces fruit yield and quality [16]. The results showed that when the nitrogen input was 300–375 kg N hm^−2^ and phosphorus input was 225–300 kg P hm^−2^, the nitrogen and phosphorus contents in the pear leaves were relatively high, and the quality of the pears was higher. The results indicated that under the current production conditions, the nitrogen input was 300–375 kg N hm^−2^ and the phosphorus input was 225–300 kg P hm^−2^, which were the most suitable nitrogen and phosphorus inputs.

### 4.2 Effects of water and fertilizer coupling on the growth, fruit yield, and quality of pears

Strategic water and fertilizer management can promote fruit tree growth. Root systems are water- and fertilizer-oriented, and root indices, such as root density and root diameter, are highly sensitive to surrounding environmental factors and nutrient supply [17]. The results of this study also showed that under the current production conditions, the appropriate water–fertilizer coupling treatments (T3, T4) were beneficial to the root growth of pear in the arid region, and the root area and root density at 0–60 cm were the highest. Meanwhile, the coupling of water and fertilizer also significantly affected the leaf growth of the pear in the arid region, and the leaf area index showed a trend of rapid rise–slow rise–slow decline during the whole growth period. This is because fruit trees grow rapidly under the influence of water and fertilizer from the flowering stage to the early stage of fruit expansion, as well as with the growth of new branches, and from the late stage of fruit expansion to fruit maturity. The leaf area index slowly declines as nutrients are transferred to the fruit and the tree leaves fall. Compared with other treatments, the leaf area index of the T3 and T4 treatments was always higher, which also indicated that suitable water–fertilizer coupling promoted the leaf growth of the pear in the arid region.

Strategic water and fertilizer management can promote fruit growth and improve fruit yield and quality. Wang et al. [18] studied the effect of water and fertilizer coupling on the growth and flowering of dwarf Fuji Apple saplings and showed through experiments that an appropriate water and fertilizer combination and management could significantly improve the nutrient status of red Fuji seedlings and promote the development of new shoots so that they could bloom and fruit in advance. According to a study by Hou et al. [19] on the effects of irrigation and fertilization mode on the growth, fruit formation process, and yield of longan, strategic water, and fertilizer management improved the growth rate of longan trees, as well as the fruit yield and fruit quality. In line with the results of previous studies, the results of this study showed that the T3 treatment was the best fit for the pear fruit shape index. The fruit yield of the pears under T3 treatment was the highest, and the primary fruit rate was as high as 78%. This was significantly higher than that of the other treatments, indicating that suitable water and fertilizer treatments were beneficial to the growth and development of the pear fruit. Suitable water and fertilizer promoted root growth, which was conducive to the transport of water and fertilizer to the ground and, therefore, promoted the growth of the pear fruit. Meanwhile, suitable water and fertilizer application can improve the leaf area index and stomatal conductance of fruit leaves [20]. This means that photosynthesis can be maintained, which is conducive to the formation of photosynthetic products and promotes fruit growth and yield.

A suitable combination of water and fertilizer can improve the fruit quality. The results of this study have shown that water and fertilizer control within a suitable range can increase the soluble solid content of the pear in the arid region, increase the soluble sugar content, reduce the titratable acid content, reduce the fruit stone cell content, and improve the fruit quality. However, insufficient or excessive water and fertilizer can reduce the fruit quality. The main reasons were as follows: the lack of water and fertilizer affected the growth of the pear fruit and then affected the accumulation of photosynthetic products and fruit quality; excessive water and fertilizer led to excessive nutrient growth of fruit trees, reduced the distribution of photosynthetic products to fruit, and affected the accumulation of photosynthetic products and fruit quality of the Korla Xiangli fruit. In conclusion, under the current production conditions, 6,300 m^3^ hm^−2^ irrigation, 375 kg N hm^−2^ nitrogen application, and 225 kg P hm^−2^ phosphorus application are the recommended water and fertilizer inputs.

## 5 Conclusions

The nutrient uptake and fruit quality of pear trees under different water, nitrogen, and phosphorus dosages were investigated comprehensively. The results showed that W3, W4, N3, N4, P3, and P4 treatments could significantly improve the nutrient utilization and fruit quality of pear trees. Therefore, W3 (6,300 m^3^ hm^−2^), W4 (6,720 m^3^ hm^−2^), N3 (300 kg N hm^−2^), N4 (375 kg N hm^−2^), P3 (225 kg P hm^−2^), and P4 (300 kg P hm^−2^) treatments were the best irrigation and fertilization scheme for the pear tree coupling test.

The coupling test of water and fertilizer showed that the irrigation amount was 6,300 m^3^ hm^−2^, the nitrogen application rate was 375 kg N hm^−2^, the phosphorus application rate was 225 kg P hm^−2^, and 0–60 cm had higher root density and root surface area, higher leaf area index, the best-fit curve of fruit shape index, and the highest gray correlation degree of fruit quality. Using the yield model prediction, the results show that 6510.06 m^3^ hm^−2^ irrigation, 337.5 kg N hm^−2^ nitrogen, and 262.5 kg P hm^−2^ phosphorus application are the best water and fertilizer applications in this area during the growth period. When the amount of water and fertilizer reached a certain limit, the fruit quality and yield were better than the amount of water and fertilizer. Under the conditions of reasonable water and fertilizer management, the yield and fruit quality of pear trees are relatively better. Therefore, improving the yield and fruit quality through rational water and fertilizer management not only meets the needs of agricultural development but also reduces the input of fruit farmers. Therefore, an irrigation amount of 6,300 m^3^ hm^−2^, a nitrogen application amount of 375 kg N hm^−2^, and a phosphorus application amount of 225 kg P hm^−2^ are recommended for local water and fertilizer treatment.
